# Rehabilitation of Edentulous Jaws with Full-Arch Fixed Implant-Supported Prostheses: An Approach with Short and Ultrashort Implants and Metal-Free Materials

**DOI:** 10.1155/2020/8890833

**Published:** 2020-07-23

**Authors:** Riccardo Aiuto, Christian Barbieri, Daniele Garcovich, Mario Dioguardi, Mattia Redaelli, Luca De Micheli

**Affiliations:** ^1^Department of Biomedical, Surgical, and Dental Science, University of Milan, Italy; ^2^Universidad Europea de Valencia, Spain; ^3^Department of Clinical and Experimental Medicine, University of Foggia, Italy; ^4^Private Practice, Albavilla, Como, Italy; ^5^Department of Periodontology, Italian Stomatological Institute, Milan, Italy

## Abstract

*Introduction*. Short implants represent a valid alternative to bone regeneration techniques. In addition, metal-free prosthetic materials have several advantages for predictable rehabilitation. This case report is aimed at illustrating the advantages of fixed implant-prosthetic rehabilitation on short and ultrashort implants and metal-free prosthetic materials. *Case report*. A 66-year-old male patient with bone atrophy was treated with temporary denture placement performed based on a rapid protocol. Once the tissues after extractions matured and aesthetic/function was studied, short implants were prosthetically placed, and a fiber-reinforced composite (FRC) bar was digitally designed for a double full-arch fixed rehabilitation. The 2-year follow-up showed the absence of peri-implantitis signs and a stable occlusal relationship of prostheses. *Discussion and conclusions*. The FRC material has excellent aesthetic properties and is low cost with a simplified and fast workflow owing to digital dentistry methods. Further studies are still needed to confirm the effectiveness of long-term therapy; however, the combination of new minimally invasive surgery and prosthetic advances seems to be very promising.

## 1. Introduction

Complete edentulism is a serious problem that affects many individuals, with different incidence and prevalence rates worldwide. Owing to advances in dentistry, the situation has improved; however, there is an increase in incidence in the elderly population. The loss of teeth without replacement with dentures implies severe loss of oral functioning [1]. Therefore, despite the progress in prevention, diagnosis, and treatment, dentists are facing the challenge of rehabilitation of patients with edentulism daily.

Sivaramakrishnan and Sridharan in their meta-analysis confirmed that oral implantology can improve the quality of life of patients with total prosthesis through the realization of implant-supported rehabilitations [2]. Total oral rehabilitation is a complex therapeutic procedure because it includes many aspects, such as the patient's emotional sphere and general health status; however, clinical difficulties are represented, for example, by atrophy of the jaw bone or limitation imposed by the presence of the inferior alveolar nerve (IAN) if endosseous implants are planned [3]. Thus, oral surgery is becoming less invasive to limit the risks and prevent excessive discomfort of the patient, who prefers to avoid bone grafts and prevent intra- and postoperative morbidity in terms of pain, swelling, bleeding, or extended operative time [4]. With a view to minimally invasive implant surgery, short implants or ultrashort implants (<6 mm in length) are a valid alternative. In a systematic review of RTCs, Amine et al. showed promising results in terms of bone loss in these types of implants that are increasingly used [5].

In addition to the surgical phase, the design of the prosthesis also plays an important role. In the past years, the interest in prosthetic dentistry has focused on composite materials, and many studies have been conducted to evaluate the performance of fixed partial dentures made of composite materials. The advantages of these composite materials over traditional metal-ceramic systems include improved aesthetics, excellent biomechanical behavior, and possibility of repair or modifying denture chairside [6]. Zaparolli et al. have recently analyzed different bar materials and manufacturing techniques, proving that fiber-reinforced composite (FRC) materials have achieved excellent functional-aesthetic and biotolerability results [7].

This case report is aimed at illustrating the advantages of fixed implant-prosthetic rehabilitation on short implants and metal-free prosthetic materials.

## 2. Case Report

**Figure 1:**
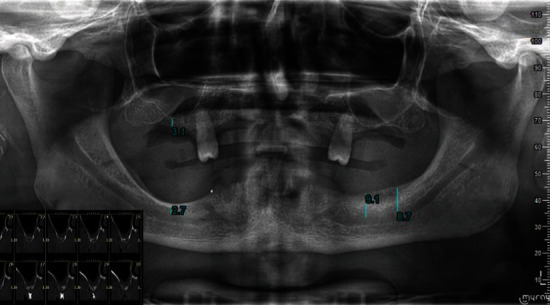
Preoperative orthopantomography and CBCT.

A 66-year-old man, with hypertension and a history of smoking, consulted us at an Italian center qualified for short dental implants. The patient had previously undergone extractions of the lower teeth and is totally edentulous, apart from the two upper second premolars. The patient had severe atrophy (Cawood and Howell classes IV and V) [8] of the jaw bones (Figure 1) and is currently wearing an incongruous mobile prosthesis. The patient requested to undergo fixed-prosthetic implant rehabilitation. In agreement with the patient, the treatment plan included extraction of remaining teeth (with 2° mobility, as described by Miller) [9], creation of two temporary upper and lower total prostheses, and finalization of the case with the prosthetically guided insertion of short implants and manufacturing of a fixed prosthesis with an FRC bar.

**Figure 2:**
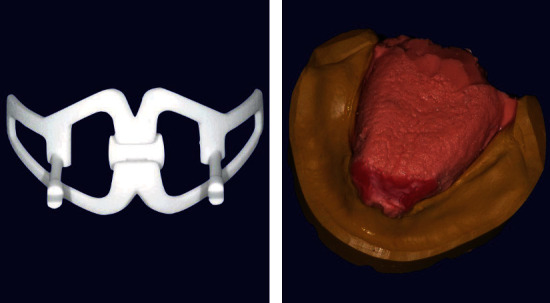
JIG BTHB device and tongue position in the model.

Four weeks after the planned extractions, the realization of total prosthesis followed a simplified protocol that started with alginate impressions (standard stainless steel trays) and detection of the craniomandibular relationships with JIG BTHB (the articulator Condylator® was then used). In the second appointment, the tooth assembly test and phonetic and aesthetic tests were performed (Figure 2). In the next visit, the prosthesis was terminated.

**Figure 3:**
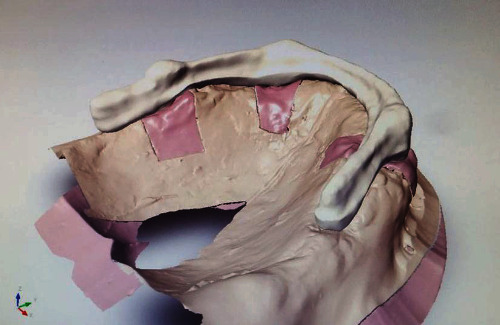
Trinia® bar CAD.

**Figure 4:**
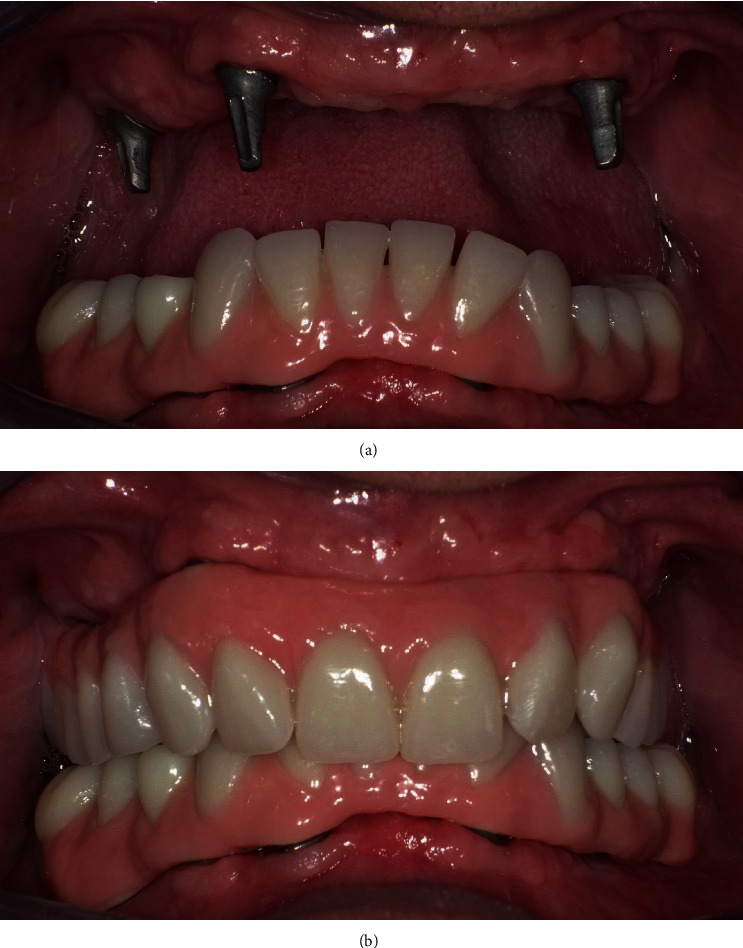
Implant abutments and full-arch fixed implant-supported prosthesis.

**Figure 5:**
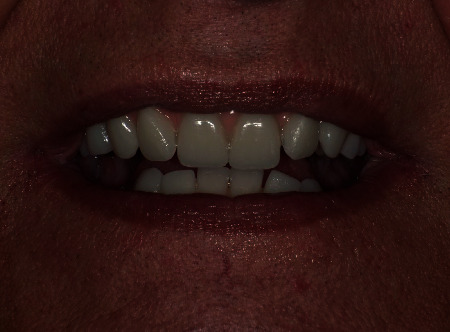
Final smile aesthetic results.

Implant surgery was performed after the maturation of the tissues, approximately 3 months after the extractions. For prosthetically guided surgery, duplicates of the prosthesis were used: in the first phase, a check was made thanks to a cone beam which showed a radiopaque material positioned inside the prosthesis; subsequently, the duplicate was drilled and used as a surgical guide. Surgical interventions involved local anesthesia with Optocain® (mepivacaine 1 : 100.000), full-thickness flap, and use of a surgical template built with the duplication of the new prostheses for implant-guided positioning. The following short implants (Bicon LLC, Boston, MA, USA) were inserted in another appointment a month later: 4.5 × 6 mm and 4.5 × 5 mm in zones 16 and 26, respectively, with transcrestal sinus lift without biomaterials and 4 × 8 mm in zone 13 and 4 × 6 mm in zone 24. In a second appointment, a few weeks later with the same modality, the lower implants were inserted in the intraforaminal region: two implants 3 × 8 mm in the areas 34 and 42 and two implants 3.5 × 8 in areas 32 and 43. The suturing material used was 4-0 Silk. The implant in zone 26 was removed after a few weeks for an osseointegration failure. Four months after the first surgical stage, the implants were uncovered, and healing abutments were placed. After 15 days of healing, the abutments were removed, and related transfers with their copings were connected and splinted to the fixtures. An implant-level transfer impression was recorded in the silicone material. The bite was recorded with articulation wax. For the realization of the prosthesis's FRC bar with Trinia® on implants, it is essential to use a partially digital workflow: not being a traditional cast bar, the design takes place thanks to a software. Trinia® CAD/CAM discs and blocks are composed of a multidirectional interlacing of fiberglass and resin in several layers. In performing this procedure, the laboratory model was digitally scanned after proving its accuracy with a light-cured resin bar for intraoral confirmation. The fiber-resin bar was digitally designed (exocad) on a computer (Figure 3). The project was performed using a milling machine operating on five axes (Roland DWX-50). The construction of the prosthesis on the bar reflected the parameters of the temporary prosthesis. The fitting of the framework was checked thanks to a radiopaque light cured resin bar for an intraoral and radiograph confirmation, and the Morse taper connection was activated. Temporary cementation of the framework was achieved (Temp-Bond, Kerr). The occlusion was evaluated and adjusted (Figures 4 and 5). The clinical protocol initially included checks every 4 months, then every 6 months. The following checks were performed: clinical examination, peri-implant probing, plaque and bleeding score, and radiographs.

## 3. Discussion

The necessary occlusal adjustments were minimal, and the patient was immediately comfortable with the new rehabilitation. He was able to chew and speak correctly, and social life and consequently psychological well-being improved. The patient was also extremely satisfied with the aesthetics achieved.

**Figure 6:**
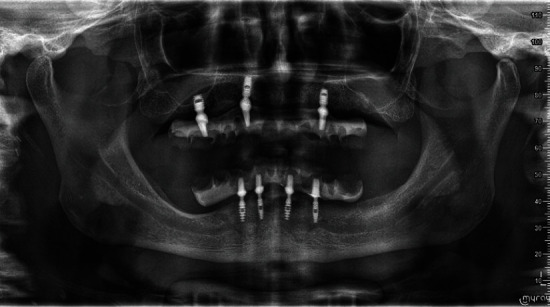
Postoperative orthopantomography at the 2-year follow-up.

Regarding the prosthetic aspect, there are important cantilevers in the distal areas of quadrants 2, 3, and 4, none of which exceeded 21 mm. As for the surgical aspect, the implant in zone 26 was lost due to lack of osseointegration before loading, but the 2-year radiographic follow-up showed the absence of signs of bone resorption around the implants (Figure 6). The fixture was characterized by a conometric connection with the abutments, both made from Ti-6Al-4V alloy, and a calcium phosphate-based treatment. A critical point for a potential microbial colonization and peri-implantitis is the connection between the implant and the prosthetic abutment [10]. The locking taper design of the implant allows a valid seal against bacteria [11].

The therapy's success was also confirmed by the absence of other clinical problems and stable and balanced occlusal relationship. In fact, the patient no longer had those discomforts that he had with the previous incongruous dentures. The patient was provided with home oral hygiene rules; in any case, he regularly returns to the clinic for periodic checks and professional oral hygiene sessions.

Complex implant-prosthetic rehabilitation, such as that described here, requires special attention from different points of view to ensure long-term clinical success. The first aspect to consider is the diagnosis and requests of the patient, who in this case preferred a fixed prosthesis. To complete the diagnosis, radiographs are fundamental: in this case of severe atrophy, in addition to orthopantomography, three-dimensional imaging techniques were also performed, which are fundamental for a correct preoperative evaluation [12].

However, it was greatly important to start from a correct design of the removable temporary prosthesis. This is to not only satisfy the aesthetic requests of the patient but also create a prosthesis respecting the neutral zone for perfect adaptation and therefore avoid dangerous forces or uncomfortable situations of poor retention. Owing to the JIG BTHB device, it was possible to perform the procedure quickly, recording the craniomandibular relationship and tongue position of the patient. In 1976, Beresin and Schiesser described the neutral zone, affirming that the soft tissues that form the internal and external boundaries of the denture space exert forces that greatly influence the stability of the dentures [13]. Therefore, the aim was to locate that area in the edentulous mouth where the teeth should be positioned so that the forces exerted by muscles will stabilize rather than unseat the denture.

A recent consensus statement also considers the importance of a balanced occlusion in an All-On-4 protocol [14]. Therefore, correct positioning of the teeth allowed the patient to not only accept the occlusal scheme but also correctly position the implants and avoid forces that cause stress on both the prosthetic and implant components. The desire for a predictable prosthesis, particularly for fixed partial dentures, led to the development of the concept of “prosthetically guided implantology.” This concept establishes the correct implant position during the diagnostic stage according to planned definitive restoration. The importance of this approach was analyzed by Tallarico and Meloni in a recent multicenter study [15].

Additionally, in this case, the use of short implants in the native bone limits the indication for tilted implants. As claimed by Scarano et al. in their study [16], the protocol with short implants avoids more invasive tissue regeneration interventions, and it can be a solution in cases of limited bone height, but it has also some limitations. In fact, an immediate load was not performed. In any case, several authors question the immediate load because, in order to immediately load an implant, primary stability is required, with a bone compression that often may cause reabsorption and replacement of nonfunctional, avascular bone [17]. This patient already had a denture and anamnesis that allowed the clinical choice for a low-risk treatment-oriented procedure.

Therefore, despite the failure of an implant, the initial prosthetic project was completed without problems. The initial program included four dental implants in maxilla, but after the loss of one of these, the patient did not want to undergo other operation for implant replacement. The choice to continue even without an implant was also dictated by the fact that occlusal contacts had been carefully studied, and a high-performance metal-free material was used.

After the analog impression, with specially splinted transfers to ensure excellent precision [18], the bar for the fixed prosthesis was designed using digital methods and is made of FRC. This material allows better distribution of occlusal loads, an advantage that contributes to the maintenance of the peri-implant bone: the implant-supported FRC not only eliminates excessive stresses in the bone-implant interface but also maintains normal physiological loading of the surrounding bone [19]. The reason for this is the lower material's flexural modulus that permits the absorption of energy from the masticatory cycle. Despite this characteristic, the performance in terms of resistance is comparable to that of other materials generally used for these purposes.

Finally, there is a biocompatibility issue. The FRC material was also found as an optimized alternative to similar materials used for the reconstruction of craniofacial bone defects [20]. Compared to metal alloys, it prevents corrosion that represents a concrete risk; for example, cobalt and nickel can be released into the oral cavity in other types of prosthesis [6]. Therefore, the advantages are multiple: the FRC material has excellent aesthetic properties and low cost with a simplified and fast workflow, owing to digital dentistry methods. Although the results are very encouraging, further studies are still needed to confirm the effectiveness of long-term therapy.

## 4. Conclusions

Rehabilitation of a patient with complete edentulism still represents a great challenge for dentists. Owing to scientific and technological advances, it is possible to implement protocols that allow less invasive implant surgery and prosthesis that correctly responds to rehabilitation needs, while respecting biology. This combination translates, for the surgical phase, into the use of short implants to reduce operative risks, limit patient discomfort, and more easily manage situations of bone atrophy that is often present in these cases. However, in the prosthetic phase, which starts from the fundamentals of the mobile prosthesis, it indicates the use of more high-performance materials, such as metal-free FRC materials, which reduce the number of implants and have great advantages in terms of durability, biotolerability, aesthetics, and treatment costs. All are facilitated by a partially digital workflow, as evidenced by the clinical case presented.
